# Discriminative validity of an abbreviated Semantic Verbal Fluency Test

**DOI:** 10.1590/1980-57642018dn13-020009

**Published:** 2019

**Authors:** José David Herrera-García, Iago Rego-García, Virginia Guillén-Martínez, María Carrasco-García, Carmen Valderrama-Martín, Rosa Vílchez-Carrillo, Samuel López-Alcalde, Cristóbal Carnero-Pardo

**Affiliations:** 1 Cognitive-Behavioral Neurology Unit, Neurology Department, Hospital Universitario Virgen de las Nieves, Granada, Spain.; 2 Neurology Department, Hospital San Cecilio, Granada, Spain.; 3 FIDYAN Neurocenter, Granada, Spain.

**Keywords:** semantic verval fluency, diagnostic utility, Alzheimer’s disease, brief cognitive test, fluência verbal semântica, utilidade diagnóstica, doença de Alzheimer, teste cognitivo breve

## Abstract

Semantic verbal fluency (SVF) is one of the most widely used tests for cognitive assessment due to its diagnostic utility (DU).

**Objective:**

our objective is to evaluate the DU to detect cognitive impairment (CI) of a short version of the SVF applied in 30 seconds (SVF_1-30_).

**Methods:**

a prospective sample of consecutive patients evaluated in a Neurology Unit between December 2016 and December 2017 were assessed with the Global Deterioration Scale (GDS), 30-second and 60-second SVF tests (animals), and the Fototest, which includes a fluency task of people’s names. The DU for CI was evaluated by the area under the ROC curve and effect size (“d” Cohen).

**Results:**

the study included 1012 patients (256 with CI, 395 with dementia). SVF_1-30_ shows a good correlation with GDS stage. The DU of SVF_1-30_ is identical to that of the classical version, applied in 60 seconds, (SVF_total_) for CI (0.89 ± 0.01; p > 0.50), and shows no significant difference for dementia (0.85 ± 0.01 vs. 0.86 ± 0.01, p > 0.15).

**Discussion:**

the DU of SVF_1-30_ is similar to that of the SVF_total_, allowing a reduction in examination time with no loss of discriminative capacity.

## RESUMO.

A fluência verbal semântica (SVF) é um dos testes mais utilizados na avaliação cognitiva devido à sua utilidade diagnóstica (UD).

**Objetivo:**

Nosso objetivo foi o de avaliar o DU de uma versão abreviada do SVF aplicado em 30 segundos (SVF_1-30_) para a detecção do comprometimento cognitivo (CC).

**Métodos:**

Amostra prospectiva de pacientes avaliados em uma Unidade de Neurologia entre dezembro de 2016 e dezembro de 2017. *Global Deterioration Scale* (GDS), um teste de SVF (animais), registrando os resultados em 30 e 60 segundos e Fototest, que inclui uma tarefa de fluência de nomes de pessoas foram aplicadas. A UD para CC foi avaliada pela área sob a curva ROC e o tamanho do efeito (“d” Cohen).

**Resultados:**

foram incluídos 1012 sujeitos (256 CC e 395 demência). O SVF_1-30_ mostrou uma boa correlação com o estágio GDS. A UD de SVF_1-30_ é idêntico ao da versão clássica (SVF_total_) para CC (0,89 ± 0,01; p > 0,50) e sem diferença significativa para demência (0,85 ± 0,01 vs. 0,86 ± 0,01; p > 0,15).

**Discussão:**

a UD do SVF_1-30_ é similar ao SVF_total_, o que permite diminuir o tempo de exploração sem perder a capacidade discriminativa.

It is estimated that more than 24 million people have dementia worldwide, and this number is predicted to double every 20 years and reach more than 80 million by 2040.^1^ Many Neurology departments are already overwhelmed by the demand from patients with some type of cognitive impairment.^2^ These disorders are more frequent in older patients, but can be presented at any age. Alzheimer’s disease (AD) is the most common dementia, and there is a need for neuropsychological instruments to facilitate its diagnosis at an early stage, allowing its early treatment and management, which increases individual, family and social benefits. These must offer high sensitivity and specificity and should be easy and quick to apply, given the limitations on clinicians’ time.

Verbal fluency tasks (VFTs) are widely used for cognitive evaluation in older adults,^3^^,^ 4 most frequently semantic VFTs. During their performance, the examiner request patients to produce as many words as possible in 60 seconds for a given category, most commonly “animals”.^5^ These tasks yield information on cognitive processes such as semantic memory, attention, working memory, inhibition, executive functions, lexical access, processing speed and cognitive flexibility.^6^^,^ 7 Semantic memory exerts the strongest influence on word adjacency in letter and semantic verbal fluency tasks. In fact, all types of fluency task scores (letter, noun, and verb) correlate with cerebral regions known to support verbal or nonverbal semantic memory.^8^ Moreover, the participation of the hippocampi and retrosplenial cortex in semantic word fluency may further explain the impaired performance of individuals with AD in semantic fluency tasks.^9^

Semantic verbal fluency is highly sensitive to brain damage, and semantic VFTs are simple, brief and easy to apply, requiring only paper, pen and watch; in addition, their sensitivity is independent of the nature of the lesion,^10^ and they can be applied to illiterate subjects. Age-and-schooling-adjusted normative, clinical and population data are available for the total number of the names generated in a minute in the Spanish language^11^^-^ 16 and age-, sex- and schooling-adjusted normative data are also available in the Portuguese language.^17^ Nevertheless, performance of this task is influenced by age and schooling, among other variables.^18^ Researchers have suggested that schooling is possibly the sociodemographic factor that have the greatest influence on verbal fluency scores.^19^^,^ 20 In addition, a less pronounced but significant effect of age compared to schooling is described in other studies.^21^^,^ 22

A semiological aspect of interest is the fact that cognitive demands may not be uniform throughout the performance of the test. Thus, the degree of cognitive effort is likely to be greater during the second half (31-60 s) than the first half of the task (0-30 s), due to the increased in demand for sustained attention, working memory and lexical search in semantic memory.^23^^,^ 24 In 2007, Fernández-Turrado et al. published normative data for the two halves of a semantic verbal fluency task (animal category) in the Spanish language; however the discriminative capacity of each half has not been analysed.^25^ In the same year, Cueto et al. described an early detection test for AD that included a 30-second SVF task;^26^ the sensitivity and specificity was reported for the whole test but not for the SVF alone. These two studies considered the 30-second semantic verbal fluency test for the first time in the Spanish language.

Two studies have analysed the production of words in the first and second halves of a 60-second verbal semantic fluency task. Fernaeus et al found that the majority of words were produced during the first half of the test and that results in the second half last 30 seconds did not increase the discriminative capacity for CI detection.^27^ In the other study, Alberca et al. examined four tasks of semantic verbal fluency with different semantic categories and found no increase in the discriminative capacity for mild CI after the first 30 seconds, recommending the use of results obtained in the first 15 or 30 seconds.^28^ However, there has been no specific analysis of the discriminative capacity for CI of a 30-second semantic verbal fluency task.

The aim of this study was to evaluate the discriminative validity of an abbreviated 30-second version of the semantic verbal fluency test to detect CI and dementia.

## METHODS

### Participants

This cross-sectional study prospectively included consecutive patients attended in a Neurology unit dedicated to Cognitive-Behavioural Neurology between December 2016 and December 2017. They had been referred to the unit for suspicion of CI by neurologists at the same centre or by general practitioners, psychiatrists, or geriatricians, based on memory complaints from the patient or their caregiver, while a small proportion were referred by the General Neurology Unit (headache, epilepsy, tremor, etc). When patients had been evaluated more than once during the study period, only the last evaluation was considered.

### Procedure and measures

The neurological examination for all patients included an abbreviated cognitive evaluation, consisting of the Fototest^29^ (including a nomination task in which the subject must name six objects shown on a slide, a 60-second verbal fluency test with people’s names and a deferred recall test in which subjects must remember objects seen at the beginning of the test), which was developed and validated in our setting,^30^ and a 30-second semantic verbal fluency test using the “animals” category (SVF_1-30_). In 79.8% of these patients, results were also recorded for the second half (SVF_31-60_) and for the whole of the 60-second SVF (SVF_total_), without modifying the instructions to patients. Results for fluency in the Fototest for names (SVF_names_) were also independently recorded.

Regardless of the results of the abbreviated evaluation, all patients referred for cognitive complaints or memory loss and all those with a total or disaggregated Fototest score below the 10^th^ percentile (≤P_10_) underwent an extensive formal cognitive evaluation (FCE) by an experienced neuropsychologist. The FCE included the assessment of orientation, attention, learning, memory, recognition, discrimination, nomination, comprehension, semantic verbal fluency, abstract thinking, calculus and executive functions, recording an abnormal performance if the result was below the 5^th^ percentile (≤P_5_) in one cognitive function or ≤P_10_ in two different functions.

The Global Deterioration Scale (GDS) stage^31^ was calculated for each patient. GDS stage 1 (GDS 1) was assigned to the subjects with no cognitive complaints, either spontaneous or in response to a direct question, and who had a normal Fototest score for their age and educational level or a normal FCE. GDS stage 2 was assigned to patients complaining of memory loss, spontaneously or in response to a direct question, and with a normal Fototest (percentile >10), and also to those with an abnormal Fototest result and normal FCE. GDS stage 3 was assigned to patients with abnormal FCE but no functional impairment in daily activities. GDS stage 4 was assigned to patients with pathological FCE and impairment of extra-domestic activities, GDS stage 5 to those with abnormal FCE and impairment of domestic (instrumental) activities and GDS stage 6 to those with abnormal ECF and impairment of basic daily life activities. No patient was assigned to GDS 7 stage.

The educational level of patients was classified as: <primary if they had not completed primary school (<6-8 years of schooling), primary if they had completed 6-8 years of schooling, and > primary if they had completed more than 6-8 years of schooling (secondary and university studies). The variability of 2 years is due to changes in the Spanish educational legislation.

### Data analysis

Descriptive analysis was conducted, with measures of central tendency (mean), dispersion (standard deviation) and distribution by percentiles. Linear regression analysis was then applied to examine the association between SVF_1-30_ and GDS stage, and the chi-square test was used for comparisons between proportions. The diagnostic utility (DU) was evaluated independently for no cognitive impairment (Non-CI) (GDS 1-2) vs. CI (GDS 3-6) and no dementia (Non-DEM) (GDS 1-3) vs. dementia (DEM) (GDS 4-6) by calculating the area under the ROC curve (AUC). The Hanley and McNeil method was used to compare the AUCs obtained for different fluency test results from the same sample. Optimal cut-points were considered those with a sensitivity (Se) >0.80 maximized by the Youden index (Se + Sp - 1), calculating the Se, specificity (Sp) and percentage of correct classifications (% CC).

## RESULTS

**Figure 1:**
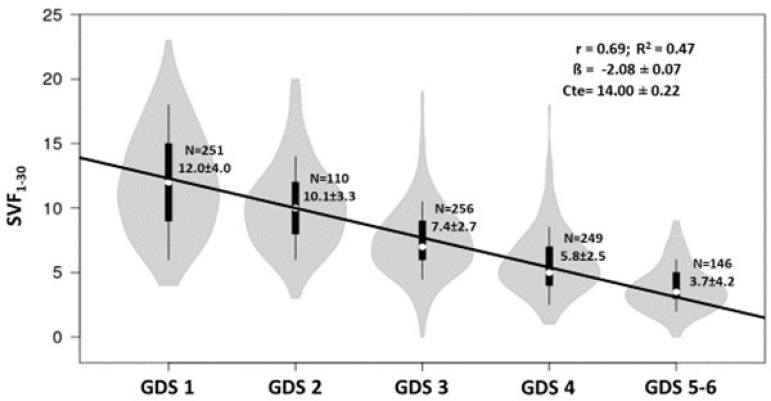
Violin graph of the distribution of SVF_1-30_ results by GDS stage.

**Figure 2:**
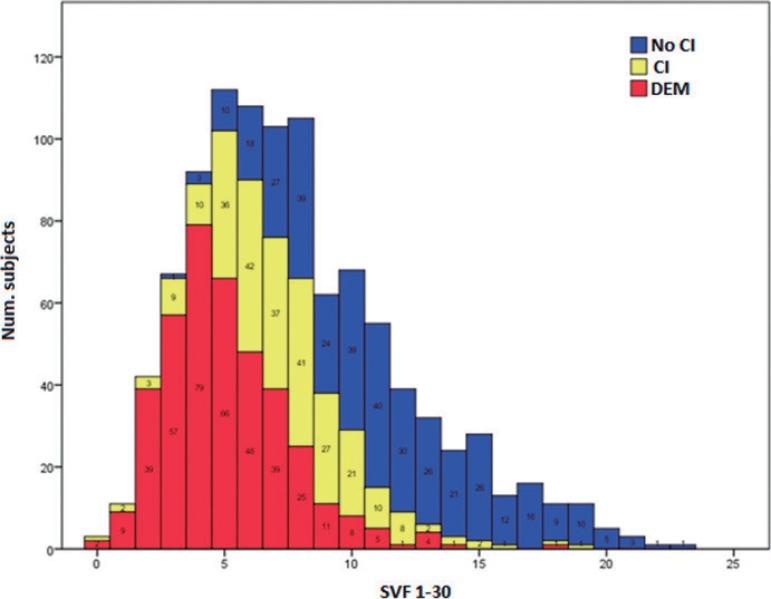
Frequency histogram of SVF_1-30_ values with indication of cognitive diagnosis (Non-CI, CI and DEM).

The study included 1012 patients, who all had Spanish as their mother tongue. Table 1 exhibits their fluency test results and sociodemographic characteristics, stratified by their cognitive diagnosis. Their ages ranged from 15 to 94 years, and 60.5% were aged over 65 years; 51.2% were female; 32.1% had not completed primary schooling and only 33.7% had completed schooling above primary level. In the multiple linear regression analysis, SVF1-30 results were associated (r 0.72, R2 0.52) with GDS stage (-1.79 ± 0.07, β ± standard error), age (-0.04 ± 0.07) and educational level (<Primary [-0.44 ± 0.23], > Primary [1.02 ± 0.23], with Primary as reference category). Figure 1 plots SVF1-30 results against GDS stage, showing a progressive reduction in the former with higher GDS stage. GDS1 was associated with a mean SVF_1-30_ score of 12.0 ± 4 (N = 251), GDS2 with score of 10.1 ± 3.3 (N = 110), GDS3 with 7.4 ± 2.7 (N = 256), GDS4 with 5.8 ± 2.5 (N = 249), and GDS5-6 with a mean SVF_1-30_ score of 3.7 ± 4.2 (N = 146). Figure 2 depicts a frequency histogram of these results with indication of the diagnosis: no cognitive impairment (Non-CI), cognitive impairment (CI) and dementia (DEM).

**Table 1:** Summary of sociodemographic characteristics and results for the total sample and stratified by cognitive diagnosis.

		No CI	CI	DEM	Total	p
No. subjects		361	256	395	1012	
Sex (women)*		177 (50.6%)	125 (50.2%)	200 (52.40%)	502 (51.2%)	n.s.
Age (years)^$^		53.5 ± 17.4	68.6 ± 11.6	73.3 ± 10.1	65.0 ± 16.1	<0.001
Age (>65 years)^$^		110 (30.5%)	192 (75.0%)	307 (78.7%)	609 (60.5%)	<0.001
Education level^#^	<Primary	59 (17.3%)	83 (34.3%)	161 (44.8%)	303 (32.1%)	
	Primary	106 (31.0%)	94 (38.8%)	122 (34.0%)	322 (34.1%)	
	>Primary	177 (51.8%)	65 (26.9%)	76 (21.2%)	318 (33.7%)	
Fototest^&^		36.5 ± 6.0	28.7 ± 3.9	21.7 ± 5.7	28.8 ± 8.4	<0.001
SVF Names^&^		20.4 ± 5.4	14.7 ± 3.7	10.8 ± 4.0	15.3 ± 6.1	<0.001
SVF (animals)	SVF_1-30_	11.4 ± 3.9	7.4 ± 2.7	5.1 ± 2.5	7.9 ± 4.2	<0.001
	SVF_31-60_^@^	5.7 ± 2.7	3.5 ± 2.1	2.2 ± 2.7	3.6 ± 2.7	<0.001
	SVF_Total_^@^	17.2 ± 5.7	10.9 ± 3.9	7.3 ± 3.8	11.4 ± 6.2	<0.001

Results are expressed as No. subjects (percentage) or mean ± s.d SVF: Semantic verbal fluency; SVF_1-30_: SVF in the first 30 seconds; SVF_31-60_: SVF between 31 and 60 seconds; SVF_Total_: SVF in one minute; No CI: no cognitive impairment; CI: cognitive impairment; DEM: dementia;

* Only registered for 981 subjects;

$ Only registered for 1007 subjects;

# Only registered for 943 subjects;

@ Only registered for 798 subjects;

& Only registered for 1010 subjects.

The words produced during SVF_31-60_ (mean of 3.6 ± 2.7 words) represented 33.7%, 32.2% and 29.6% of the SVF_total_ for Non-CI, CI and DEM patients respectively. This production is lower than the percentages for the mean number of words produced by these patients during SVF_1-30_ (mean of 11.4 ± 6.2 words): 66.3%, 67.8% and 70.4%, respectively These percentages did not significantly differ among the three diagnostic categories (χ^2^ = 1.47, p = 0.23, for the greatest difference).

Table 2 displays the Se, Sp, Youden index and %CC values for the optimal cut-off points in the different fluency tests to obtain Se >0.80. A cut-off SVF_1-30_ score of 8/9 correctly discriminated CI from non-CI in 80.70% of the subjects, highly similar to the percentage correctly classified as CI/non-CI using a SVF_total_ cut-off score of 12/13 (χ^2^ = 0.14, p = 0.71). There were also no significant differences in the correct classifications for DEM with cut-off points of 7/8 for SVF_1-30_ and 12/13 for SVF_total_ (73.93% vs 75.69%, p = 0.41).

**Table 2:** Parameters of diagnostic utility for CI and DEM for the best cut-off points on the different semantic verbal fluency tests.

	CI						DEM				
	PdC	S	E	Y	%CC		PdC	S	E	Y	%CC
SVF_1-30_	8/9	0.83	0.75	0.58	80.70		7/8	0.85	0.66	0.51	73.93
SVF_31-60_	4/5	0.83	0.63	0.46	76.82		4/5	0.90	0.47	0.37	64.54
SVF_Total_	12/13	0.81	0.76	0.57	79.95		10/11	0.82	0.71	0.53	75.69
SVF_Names_	16/17	0.83	0.75	0.58	80.65		14/15	0.83	0.71	0.54	76.13

SVF: Semantic verbal fluency; SVF_1-30_: SVF in the first 30 seconds; SVF_31-60_: SVF between 31 and 60 seconds; SVF_Total_: SVF in one minute; SVF_Names_: SVF for naming task in Phototest.

Table 3 displays the normative SVF1-30 scores stratified by age and educational level.

**Table 3:** Normative values of abbreviated verbal semantic fluency, stratified by age and educational level in subjects without CI.

	< 65 years				> 65 years		
	< Primary	Primary	> Primary		< Primary	Primary	> Primary
No. of subjects	23	70	144		36	36	33
x±sd	9.1±3.0	11.2±3.6	13.5±3.7		8.1±2.2	9.5±2.6	10.2±3.8
–1.0z	6	8	10		6	7	6
–1.5z	5	6	8		5	6	5
–2.0z	3	5	6		4	4	3
P_10_	5	7	8		6	6	6
P_5_	4	6	7		5	5	4

**Figure 3:**
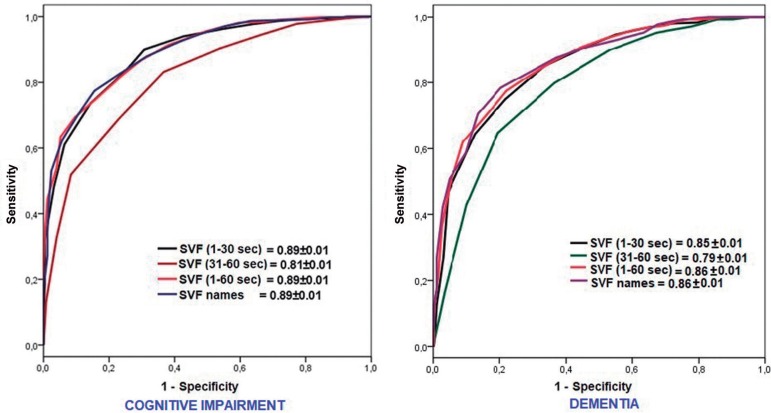
ROC curves of the different semantic verbal fluency tests for cognitive impairment and dementia. The figures correspond to the area under the ROC curve ± standard error.

The DU of SVF_1-30_ was identical to those of the SVF_total_ and SVF_names_ for CI (0.89 ± 0.01; p > 0.50) and did not significantly differ from those for DEM (0.85 ± 0.01 vs. 0.86 ± 0.01; p > 0.15). The DU of SVF_31-60_ was significantly lower in both cases (0.81 ± 0.01 [p < 0.001] and 0.79 ± 0.001 [p < 0.01], respectively) (Figure 3).

## DISCUSSION

The 30-second SVF task (“animal names”) showed a strong inverse correlation with the cognitive state of these patients, observing a decreased score with higher GDS stage. More than two-thirds of the words were produced within the first 30 seconds of the classical SVF task (66.8% for CI patients and 70.4% for DEM patients), while the second 30-second period only contained the remaining third of the words produced. These findings are in agreement with the results published by Fernaeus et al.^24^ and Alberca et al.^26^; however, unlike in the present study, they did not compare the DU between the first and second halves of the test.

Our main finding was that the short SVF_1-30_ had the same DU as the classical 60-seconds SVF. The discriminative capacity of the 60-second SVF was lower for the second half (SVF_31-60_) than for the first half and did not add value to the DU of the SVF_total_. Hence, the discriminative capacity of the classic SVF task depends on the words produced by subjects during the first 30 seconds, and their performance during the second half of the test adds no further DU.

In the short SVF task, the cut-off score for a sensitivity of 80% was 8/9 words, which discriminated between subjects with and without cognitive impairment in 80.7% of cases. This cut-off point was very similar that obtained for the DU of the SVF_total,_ confirming that SVF_1-30_ represents the discriminative part of the classical task.

Given that no added benefit is obtained by extending the duration of the SVF to 60 seconds, it appears rational to limit the test to 30 seconds. This would allow clinicians more time for other cognitive examinations, increasing the precision of the diagnosis, or for other clinical activities. The total consultation time saved in the present series of patients adds up to almost 8 ½ hours. Limitation of the tests to the first 30 seconds would be especially useful when clinical resources are limited and there is a high demand.

Although this study was conducted using animals as the sole semantic category, similar results could be expected using other categories; however, further research is required to verify this assumption. The “animals” category is one of the most widely used and can be readily applied to illiterate subjects. Further potential limitations are the single-centre study design and also the predominantly low educational level of the sample, although this is representative of the elderly population in Spain, who generally had limited access to education in their youth. Study strengths include the large size of the sample (N = 1012) and its naturalistic character, given that study enrolment was prospective, systematic, and consecutive. In addition, all patients showing some degree of cognitive impairment underwent standard cognitive assessment by a neuropsychologist.

In conclusion, the VFT (animals) performed in 30 seconds has the same diagnostic utility for cognitive impairment and dementia as the classical 60-second test. Hence, extension of the test beyond the first 30 seconds does not appear to be reasonable, efficient or practical.
